# Social Isolation-Induced Territorial Aggression in Male Offspring Is Enhanced by Exposure to Diesel Exhaust during Pregnancy

**DOI:** 10.1371/journal.pone.0149737

**Published:** 2016-02-26

**Authors:** Satoshi Yokota, Shigeru Oshio, Nozomu Moriya, Ken Takeda

**Affiliations:** 1 The Center for Environmental Health Science for the Next Generation, Research Institute for Science and Technology, Tokyo University of Science, Noda, Chiba, Japan; 2 Department of Hygiene Chemistry, School of Pharmaceutical Sciences, Ohu University, Koriyama, Fukushima, Japan; 3 Department of Biopharmaceutics, Hyogo University of Health Sciences, Kobe, Hyogo, Japan; Hasselt University, BELGIUM

## Abstract

Diesel exhaust particles are a major component of ambient particulate matter, and concern about the health effects of exposure to ambient particulate matter is growing. Previously, we found that *in utero* exposure to diesel exhaust affected locomotor activity and motor coordination, but there are also indications that such exposure may contribute to increased aggression in offspring. Therefore, the aim of the present study was to test the effects of prenatal diesel exhaust exposure on social isolation-induced territorial aggression. Pregnant mice were exposed to low concentrations of diesel exhaust (DE; mass concentration of 90 μg/m^3^: DE group: n = 15) or clean air (control group: n = 15) for 8 h/day during gestation. Basal locomotion of male offspring was measured at 10 weeks of age. Thereafter, male offspring were individually housed for 2 weeks and subsequently assessed for aggression using the resident−intruder test at 12 weeks of age, and blood and brain tissue were collected from the male offspring on the following day for measuring serum testosterone levels and neurochemical analysis. There were no significant differences in locomotion between control and DE-exposed mice. However, DE-exposed mice showed significantly greater social isolation-induced territorial aggressive behavior than control mice. Additionally, socially-isolated DE-exposed mice expressed significantly higher concentrations of serum testosterone levels than control mice. Neurochemical analysis revealed that dopamine levels in the prefrontal cortex and nucleus accumbens were higher in socially isolated DE-exposed mice. Serotonin levels in the nucleus accumbens, amygdala, and hypothalamus were also lower in the socially isolated DE-exposed mice than in control mice. Thus, even at low doses, prenatal exposure to DE increased aggression and serum testosterone levels, and caused neurochemical changes in male socially isolated mice. These results may have serious implications for pregnant women living in regions with high levels of traffic-related air pollution.

## Introduction

Diesel exhaust particles (DEPs), which are derived from diesel exhaust (DE) engines [1], are among the most abundant air pollutants in urban environments. In 2012, the International Agency for Research on Cancer, part of the World Health Organization, classified DE as carcinogenic to humans based on evidence that DE exposure is associated with an increased risk of lung cancer [2, 3].

Particulate matter is divided into the following categories by size: ultrafine (aerodynamic diameter < 100 nm), fine (< 2.5 μm), and coarse (< 10 μm). Particulate matter present in the atmosphere mostly comprises ultrafine particles [4, 5], which can disperse over long distances and can remain suspended in ambient air for long periods of time; thus, humans can be significantly exposed to ultrafine particles via inhalation [6]. Ultrafine particles are easily internalized and then induce cellular responses [7]. Once inhaled, ultrafine particles can enter the circulatory system and accumulate in various tissues, including the brain [8, 9]. Therefore, ultrafine particles may have greater toxic effects on the central nervous system, particularly in structures such as the olfactory bulb, cerebral cortex, and hippocampus, than larger particulate matter [10, 11]. Metal elements of DEPs that contained nano-sized particles were found in the olfactory epithelium and olfactory bulb of mice exposed to DEPs in an inhalation chamber for 1 month [12]. We have previously reported that maternal exposure to DE caused accumulation of DEP-like substances in the brains of the offspring, and that these particles induced apoptosis [13]. Furthermore, prenatal exposure to DE and DEPs causes behavioral dysfunction, such as leaning/memory and motor-coordination deficits, in male mice [14–16]. However, these studies have typically used higher exposures to DE than are encountered in most environments, and the effect of more typical exposures remains unclear.

Many chemicals released into the environment act as endocrine disruptors by mimicking the action of estrogen. Previous studies have found that plasma testosterone levels are increased by both chronic exposure to DE [17] and maternal exposure to DE [18]. Increased testosterone levels may result in estrogen-related aggression, as testosterone is converted to estradiol, which acts on estrogen receptors, by the enzyme aromatase. In fact, most effects of testosterone on aggression occur after aromatization [19]. Overall, these results suggest a causal link between prenatal exposure to DE and increased aggression; however, such a relationship has not yet been shown experimentally.

Territorial aggressive behavior in rodents reared in social isolation (defined as “residents”) is induced by exposure to an intruder (resident−intruder interaction). An encounter with an intruder can cause neurobiological changes that underlie aggressive behaviors in the resident, as has been shown by several neurochemical studies. For instance, encounters with an intruder have been found to increase dopamine (DA) levels in the prefrontal cortex and nucleus accumbens, and to decrease serotonin (5-HT) levels in the cortex of the resident rat [20]. Aggressive encounters have also been found to increase phasic DA transmission in the mesolimbic pathway in rats that experienced social defeat [21].

In this study, we used the resident−intruder model to determine the effects of prenatal exposure to low doses of DE on social isolation-induced territorial aggressive behavior in male offspring.

## Results

### Characterization of DE

**Fig 1:**
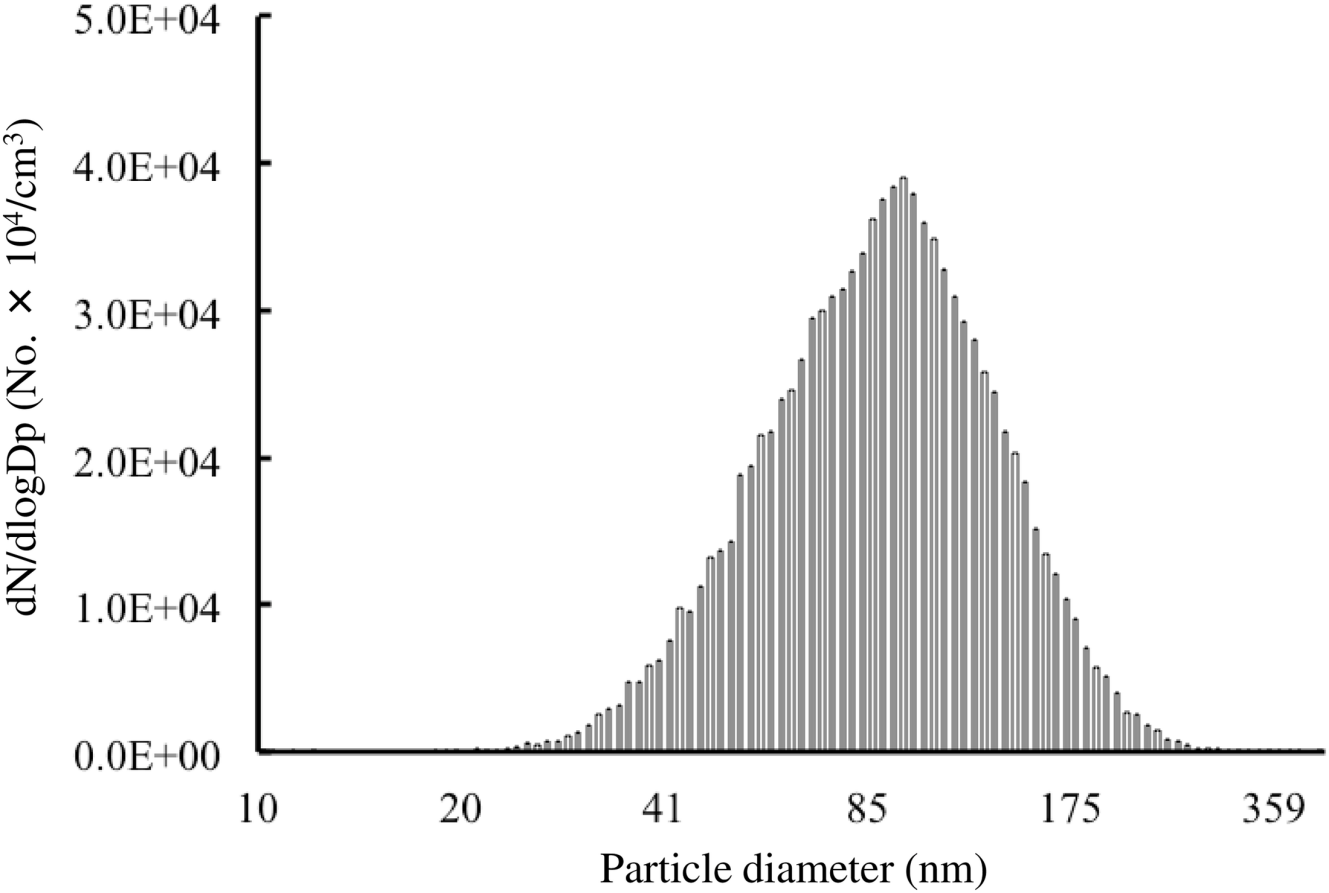
Characterization of diesel exhaust particles. Distribution of the size of diesel exhaust particles (10–410 nm) derived from a diesel exhaust engine.

The diameter distribution of DEPs in the DE chamber peaked at 94.7 nm (Fig 1). The average concentration of DEPs was, on average, 8.4 ± 1.2 × 10^4^ particles/cm^3^. The average mass concentration of these exhaust constituents was maintained at 90 μg/m^3^ (2.10 ppm for CO, 0.131 ppm for NO_2_, and less than 3.62 × 10^−3^ ppm for SO_2_) for the exposure experiments.

#### Effects of prenatal DE exposure on litter size, body weight, and locomotor activity of male offspring

There were no significant differences in litter size between the control and diesel exhaust-exposed (DE) groups (control: 11.8 ± 1.6; DE: 11.6 ± 2.0). The body weight of male offspring was also not affected by maternal DE exposure (postnatal day 1: control 2.0 ± 0.2 g; DE 2.1 ± 0.3 g; 12 weeks: control 44.4 ± 1.2 g; DE 44.7 ± 1.6 g). No deaths or malformations were observed in either the control or DE-exposed mice.

**Fig 2:**
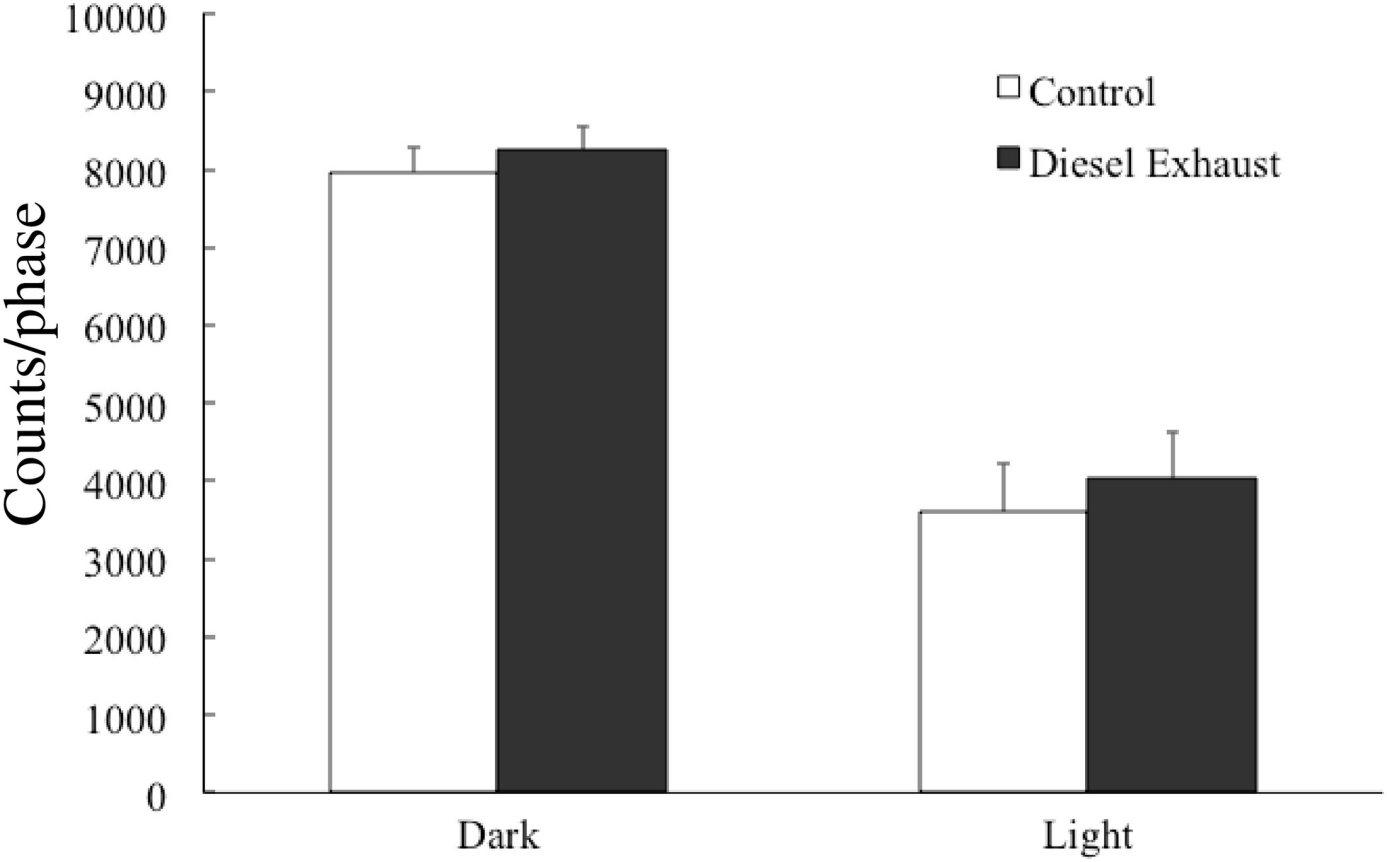
Effects of prenatal exposure to diesel exhaust (DE) on spontaneous locomotor activity. Data are expressed as total spontaneous motor activity in control and DE-exposed mice. There were no differences between the control and DE-exposed mice in terms of spontaneous motor activity. Each column represents the mean total activity count ± SEM for 15 mice.

We assessed the basal behavior of mice by measuring spontaneous motor activity. There were no significant differences between control and DE-exposed mice in locomotion during both light and dark cycles (Fig 2).

#### Effects of prenatal DE exposure on aggression in male resident offspring

**Fig 3:**
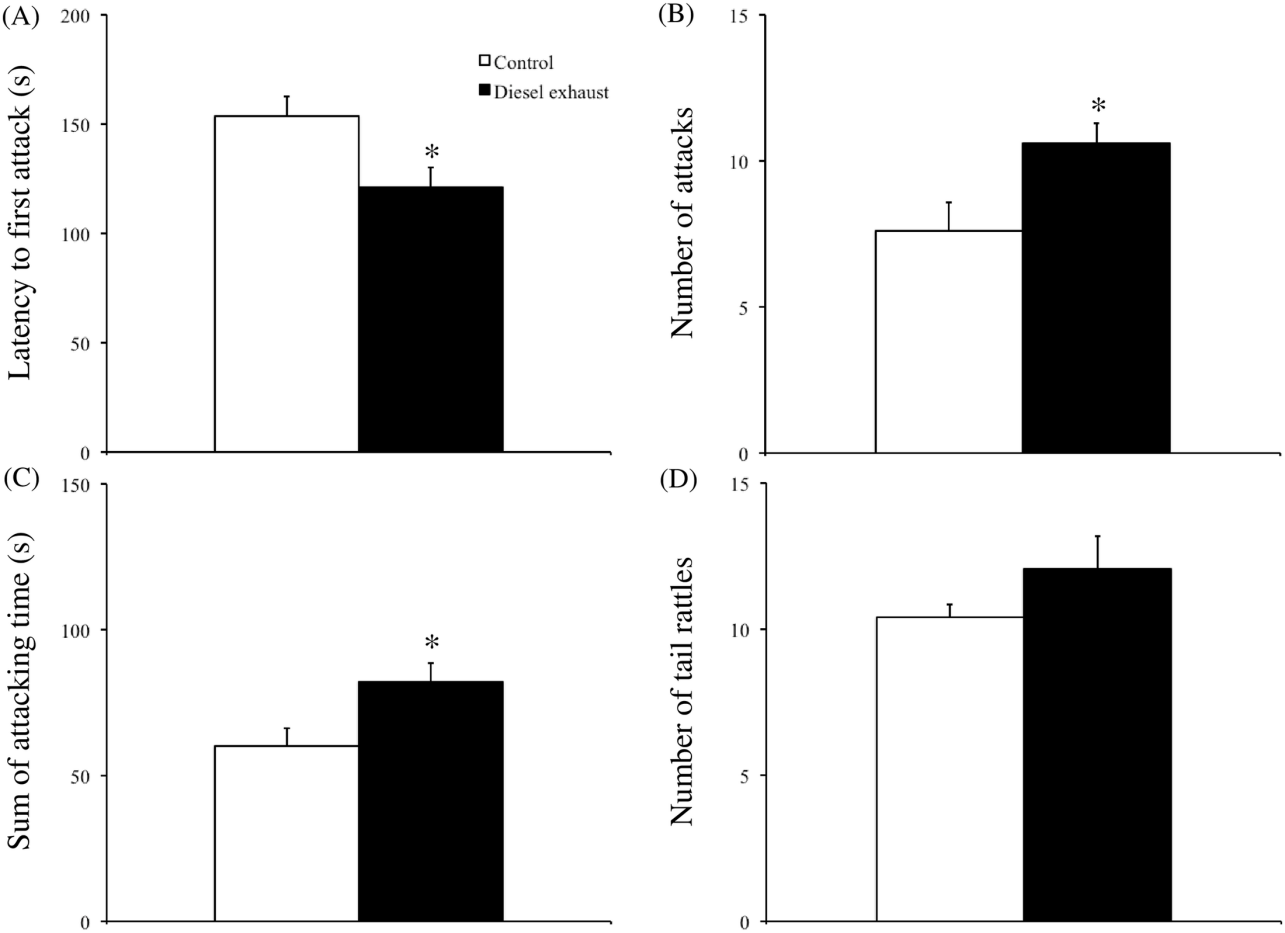
Effects of prenatal exposure to diesel exhaust (DE) on social isolation-induced territorial aggressive behavior. Social isolation-induced territorial aggressive behavior was measured using the resident intruder test. We measured (A) the latency to the first attack (sec), (B) the number of attacks, (C) the sum of attacking time, and (D) the number of tail rattles. Each (white: control, black: DE) column represents the mean ± SEM for 15 mice. *P < 0.05, Student’s *t*-test.

To examine the territorial aggression of residents (in both the control and DE-exposed groups) at 12 weeks of age, we performed a resident−intruder test. Fig 3 shows the resident males’ aggressive behavior within the 10-min test period. DE-exposed residents displayed a significantly shorter attack latency than control residents (P < 0.05, Fig 3A). Additionally, DE-exposed residents attacked more often (P < 0.05, Fig 3B) and spent more time in attacks (P < 0.05, Fig 3C) than control residents. However, the number of tail rattles in DE-exposed residents was similar to that in control mice.

#### Effects of prenatal exposure to DE on serum testosterone levels of male residents

**Fig 4:**
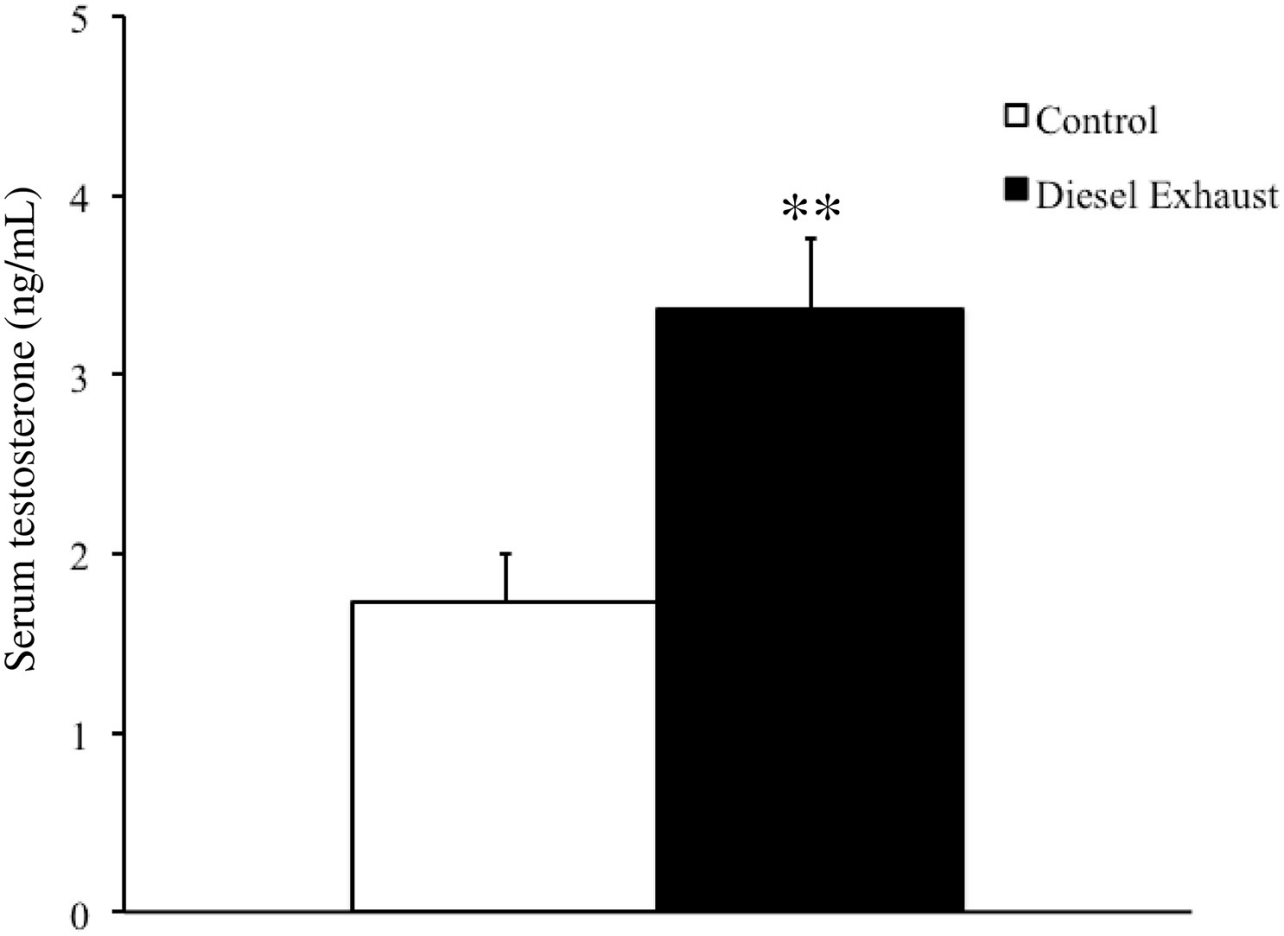
Effects of prenatal exposure to diesel exhaust (DE) on serum testosterone levels. Serum testosterone levels were quantified. Each (white: control, black: DE) column represents the mean ± SEM for 15 mice (*P < 0.05, Student’s *t*-test).

To investigate the cause of increased aggression, we measured serum testosterone levels in both control and DE-exposed residents. After the resident−intruder test, serum testosterone levels of DE-exposed residents were approximately 2-fold higher than those in control residents (1.7 ± 0.3 ng/mL vs. 3.4 ± 0.4 ng/mL, respectively; P < 0.01: Fig 4).

#### Effects of prenatal exposure to DE on the dopaminergic and serotonergic systems of male residents

**Fig 5:**
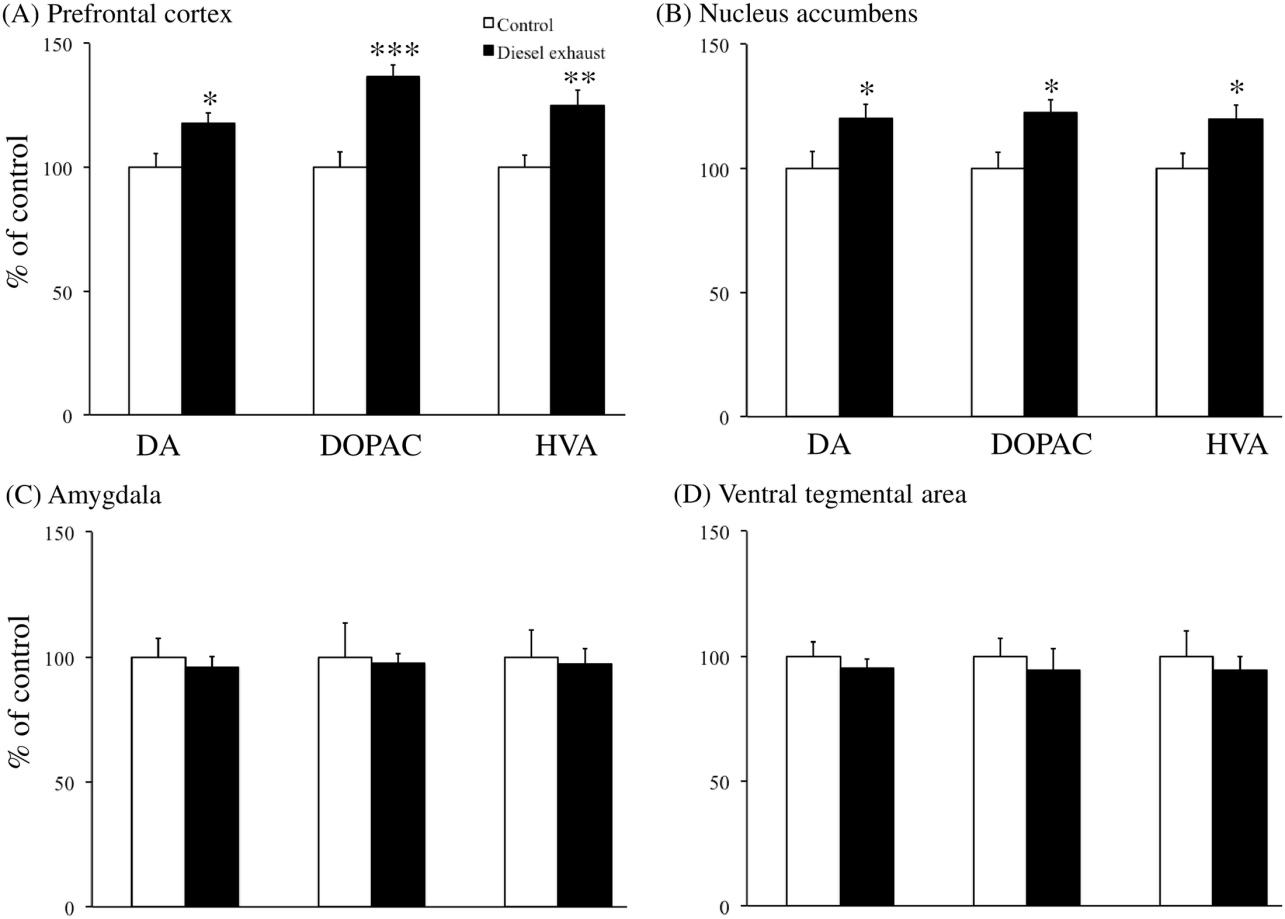
Effects of prenatal exposure to diesel exhaust (DE) on the dopaminergic system. Levels of dopamine (DA) and its metabolites (DOPAC and HVA) were analyzed (A) in the prefrontal cortex, (B) in the nucleus accumbens, (C) in the amygdala, and (D) in the ventral tegmental area. Each column (white: control, black: DE) represents the mean ± SEM for 15 mice (*P < 0.05 by Student’s *t*-test).

Brain samples were obtained after the resident−intruder test, and the levels of DA, 5-HT, and their metabolites were examined in various brain regions. Prenatal exposure to DE changed the levels of DA and its metabolites 3,4-dihydroxyphenylacetic acid (DOPAC) and homovanillic acid (HVA) in the prefrontal cortex and nucleus accumbens of male resident offspring compared to control mice. Specifically, *in utero* exposure to DE increased the levels of DA (P < 0.05, Fig 5A), DOPAC (P < 0.001, Fig 5A), and HVA (P < 0.01, Fig 5A) in the prefrontal cortex and in the nucleus accumbens (P < 0.05, Fig 5B). There were no significant differences in the levels of these factors in the amygdala and ventral tegmental area between control and DE-exposed resident offspring (Fig 5C and 5D).

**Fig 6:**
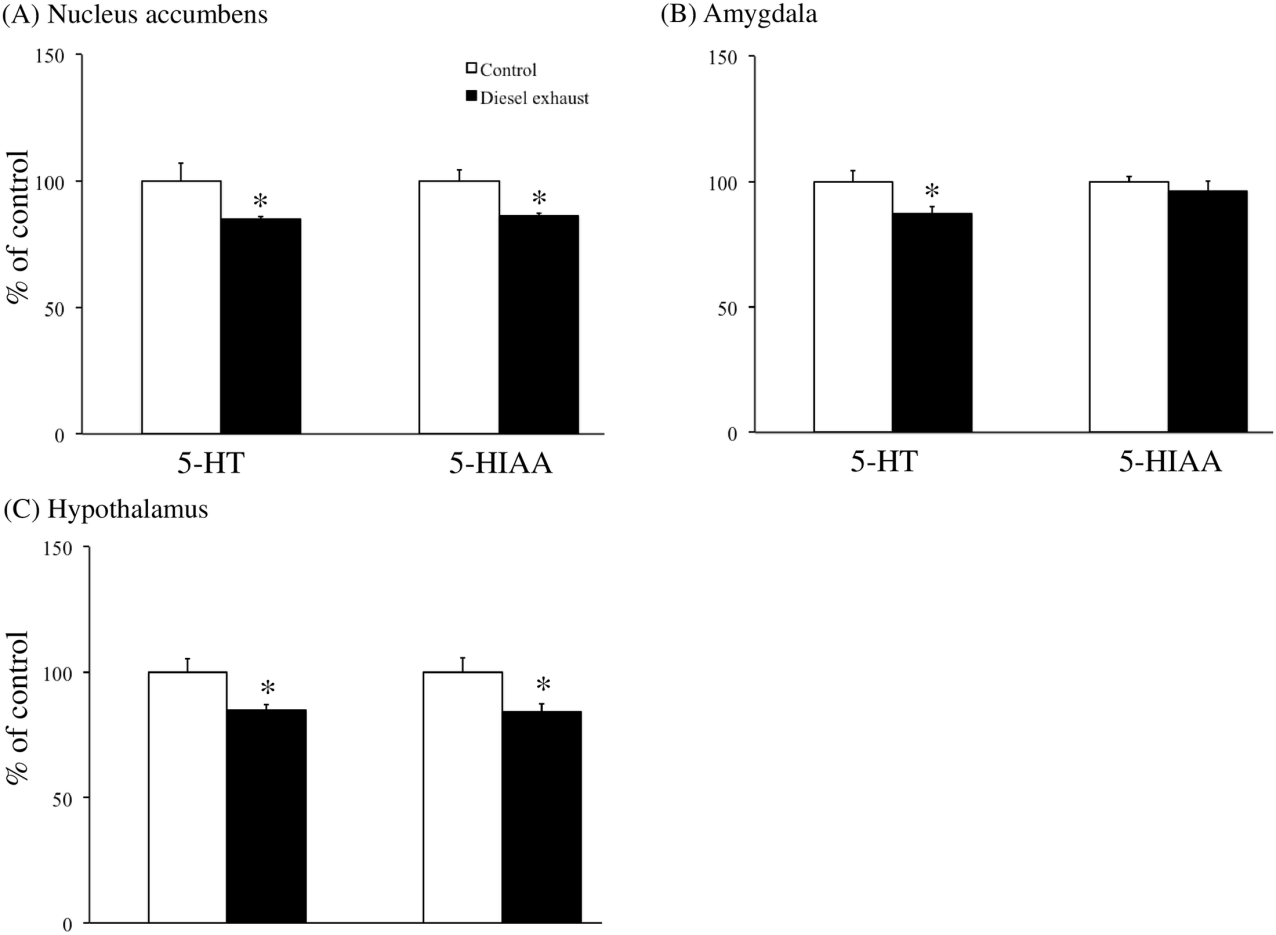
Effects of prenatal exposure to diesel exhaust (DE) on the serotonergic system. Levels of serotonin (5-HT) and 5-hydroxyindole-3-acetic acid (5-HIAA) (A) were analyzed in the nucleus accumbens, (B) in the amygdala, and (C) in the hypothalamus. Each column (white: control, black: DE) represents the mean ± SEM for 15 mice (*P < 0.05, Student’s *t*-test).

Prenatal exposure to DE also changed the levels of 5-HT and its metabolite 5-hydroxyindole-3-acetic acid (5-HIAA) in the nucleus accumbens, amygdala, and hypothalamus of male resident offspring, as compared to control mice. Specifically, *in utero* exposure to DE resulted in a lower concentrations of 5-HT and 5-HIAA (both P < 0.05, Fig 6A) in the nucleus accumbens compared to that in control mice. *In utero* exposure to DE also reduced the levels of 5-HT (P <0.05, Fig 6B) in the amygdala compared to that in control mice. Finally, *in utero* exposure to DE resulted in lower concentrations of 5-HT and 5-HIAA (both P < 0.05, Fig 6C) in the hypothalamus. There were no significant differences in the serotonergic systems of the prefrontal cortex and brainstem between control and DE-exposed resident offspring (data not shown).

## Discussion

The present study has demonstrated for the first time that exposure to even relatively low mass concentrations of DE (90 μg/m^3^) during pregnancy affects the behavior and central nervous system of male offspring. In particular, maternal DE inhalation caused more territorial aggressive behavior in residents and subsequent neurochemical changes in various brain regions of their offspring, as compared to control mice.

These findings may have important implications for humans. Indeed, accumulating evidence indicates that exposure to particulate matter in the air can induce neuroinflammation and oxidative stress that may trigger neurodegenerative disorders, such as Alzheimer’s and Parkinson’s disease [22–26]. However, no evidence concerning the relationship between exposure to particulate matter in air pollution and increased aggressive behavior has yet been reported, except for a study reporting the effects of prenatal cigarette smoke exposure [27], which increased the number of attacks and altered DA and 5-HT levels in the striatum and prefrontal cortex. The present study highlights the importance of considering the effects of *in utero* exposure to DE on social isolation-induced territorial aggressive behavior in the next generation.

Previous studies have shown that territorial aggressive behavior in rodents is influenced by the rearing environment. In particular, it has been shown in different strains of rodents that greater territorial aggression occurs after the rodents experience social isolation [28–31]. A relationship between social isolation and brain monoamine levels has been reported [32–38]; changes in the monoaminergic activity of mice cause them to exhibit territorial aggression [39–42]. However, to date, the effects of maternal exposure to DE on territorial aggressive behavior and subsequent changes in brain dopaminergic and serotonergic systems of socially isolated resident offspring have been left unexplored. Here, for the first time, we have shown that *in utero* DE exposure results in greater and more pronounced territorial aggressive behavior in such male offspring.

We also found that maternal exposure to DE resulted in higher levels of DA and its metabolites (DOPAC, HVA) in the prefrontal cortex and nucleus accumbens of socially isolated male offspring than in control mice. From weaning at postnatal day 21, long-term social isolation of rats increases basal DA levels in the prefrontal cortex and nucleus accumbens [34]; however, no such changes are observed when social isolation is initiated in rats older than 7 weeks [37]. Therefore, social isolation in the present study was initiated when mice (both control and DE-exposed male offspring) were 10–12 weeks of age. Consistent with previous studies, we verified in the present study that social isolation *per se* did not affect dopaminergic systems by comparing DA levels in various brain regions of a group-housed control with those of mice reared in isolation for 2 weeks (group housing: 98±6.0%, isolation: 100±5.6% in the prefrontal cortex; group housing: 101±6.9%, isolation: 100±6.6% in the nucleus accumbens; group housing: 100±7.5%, isolation: 100±7.3% in the amygdala; group housing: 99±5.1%, isolation: 100±5.8% in the ventral tegmental area). Neurochemical analysis of dopaminergic systems showed that *in utero* exposure to DE resulted in increased susceptibility to social isolation-induced changes in DA and its metabolites (DOPAC, HVA), which may be related to the greater degree and incidence of territorial aggressive behavior.

We showed that prenatal exposure to DE resulted in lower 5-HT levels in the nucleus accumbens, amygdala, and hypothalamus than in control mice. Serotonergic systems are known to be involved in territorial aggression; for example, socially isolated mice show increased territorial aggression when brain tryptophan levels (which contribute to 5-HT synthesis) are decreased [39]. Additionally, 5-HT1B receptor-knockout resident mice have been shown to attack intruder mice more quickly with a greater number of attacks than wild-type residents [40]. Similarly, treatment with 5-HT1A and 5-HT1B receptor agonists has been found to reduce territorial aggression [41, 42]. Together, these findings suggest that the greater territorial aggression exhibited by DE-exposed resident male offspring may result from enhancement of social isolation-induced serotonergic system dysfunction.

However, it remains unclear why DE exposure would increase territorial aggression via serotonergic dysfunction. One possible consideration is that DE contains estrogenic and anti-estrogenic compounds [43]. Several studies have related estrogen to aggressive behavior; for instance, prenatal exposure to estrogenic chemicals increases territorial aggressive behavior in mice [44], transgenic male mice deficient in estrogen receptors display fewer aggressive behaviors [45, 46], and estrogenic metabolites have been found to modulate the ability of serotonin 5-HT1A and 5-HT1B agonists to inhibit aggression [47]. Therefore, estrogenic or anti-estrogenic chemical substances that are present in DE may modulate territorial aggressive behaviors by acting on 5-HT1 receptors.

The present study also found that *in utero* exposure to DE increased serum testosterone levels in socially isolated resident offspring. Testosterone and estrogen are closely related; estrogen is produced by circulating testosterone via the enzyme aromatase. Testosterone has also been found to change the action of 5-HT1A and 5-HT1B agonists on male aggression [48]. We have previously reported that serum testosterone levels of male offspring are significantly higher than those of controls after *in utero* high-dose exposure to DE (DEP mass concentrations of 1000 μg/m^3^ and 3000 μg/m^3^), but not after *in utero* low-dose exposure to DE (DEP mass concentration of 300 μg/m^3^) [21]. However, we showed here that even low-dose exposure to DE (90 μg/m^3^) during pregnancy increased serum testosterone levels of socially isolated resident offspring. Social isolation of mice has been previously reported to increase serum testosterone levels [49]. Taken together, these results suggest that DE may enhance the social isolation-induced elevation of testosterone levels.

Finally, in the present study, maternal exposure to low DE concentrations did not affect the spontaneous motor activity of the male offspring. Previous studies have shown that *in utero* exposure to higher concentrations of DE significantly reduces locomotion of male offspring [14, 50]. Recently, the effects of *in utero* DE inhalation on the central nervous system of offspring have also been reported [14, 15, 50–52]. However, the majority of these studies used substantially higher exposure levels than that encountered in the natural environment. The present study addressed this issue by using the lowest mass concentration of DE reported to date. We have demonstrated that even low-dose prenatal DE exposure affects territorial aggressive behavior and some parameters known to be involved in territorial aggression, such as DA and 5-HT levels in the brain and testosterone levels in the serum.

The Environmental Protection Agency has set the National Ambient Air Quality Standards for particle pollution criteria [53], viz., a mass concentration of fine and coarse particles of 35 μg/m^3^ and 150 μg/m^3^ per day for fine and coarse particles, respectively. Individuals living near roads and underground mines have a higher exposure to particulate matter than these environmental standards, and may thus be subjected to the toxic effects of these particles [54]. Future investigations on the effects of *in utero* exposure to particulate matter on the central nervous system of the next generation are needed to determine the exact level at which no adverse effects are observed both in both animal and human experiments, by determining the effects of exposure to even fewer particles at a lower mass concentration than that used in the present study. Further studies should also investigate the relationship between low-dose DE exposure and the onset of cranial nerve disease, and should clarify the effects of low-dose DE exposure on the central nervous systems.

## Conclusions

The present study used the lowest mass and particle concentration among DE studies to date to investigate whether *in utero* exposure to DE affects the central nervous systems of the next generation. Despite using such a low-dose exposure, we found that prenatal exposure to DE increased aggressive behavior in male offspring mice. Additionally, compared to controls, we identified higher levels of DA and its metabolites in the prefrontal cortex and nucleus accumbens, and lower levels of 5-HT and its metabolite in the nucleus accumbens, amygdala, and hypothalamus of mice that had been exposed to low doses of DE prenatally. Finally, even at this low dose of DE exposure during pregnancy, male offspring had higher serum testosterone levels than control mice. These results may have serious implications for cognitive effects in children of mothers exposed to elevated air pollution levels, such as that seen in the vicinity of roads and underground mines. Our study highlights the need for reducing or even preventing particulate matter exposure to protect pregnant women and their children from the consequent adverse health effects.

## Materials and Methods

### Diesel exhaust

As described in detail previously [55], diesel exposure was achieved and characterized. Briefly, a four-cylinder 2,179 cc diesel engine (Isuzu Motors Ltd., Tokyo, Japan) was operated at a speed of 1500 rpm and 80% load with diesel fuel. The exhaust was introduced into a stainless steel dilution tunnel (216.3 mm diameter × 5250 mm) where it was mixed with clean air. Particle size distributions (range: 10–410 nm) were investigated using a scanning mobility particle-sizer apparatus (model 3936, TSI Inc., St. Paul, MN, USA) consisting of a condensation particle counter (model 3785, TSI Inc.) and a differential mobility analyzer (model 3081, TSI Inc.). The apparatus was operated at a sample flow rate of 0.6 L/minute and a sheath flow rate of 6.0 L/minute. The mass concentration of DEPs was measured using a Piezobalance Dust Monitor (model 3521, Kanomax, Inc., Osaka, Japan). The concentration of DEPs was measured using a condensation particle counter (model 3007, TSI Inc.), which can count particles with diameters in the range of 10–1000 nm. Concentrations of gas components (i.e., nitric oxide [NO_x_], sulfur dioxide [SO_2_], and carbon monoxide [CO]) in the chambers were measured using an NO-NO_2_-NO_x_ analyzer model 42i (Thermo Fisher Scientific Inc., Franklin Lakes, MA, USA), Enhanced Trace Level SO_2_ Analyzer Model 43i-TLE (Thermo Fisher Scientific Inc.), and CO Analyzer, model 48i (Thermo Fisher Scientific Inc.), respectively.

### Animals and maternal exposure

All experiments were performed in accordance with National Institutes of Health (NIH, USA) guidelines for animal experiments and were approved by Tokyo University of Science’s Institutional Animal Care and Use Committee.

Thirty pregnant ICR mice at gestational day 1, obtained from SLC Co. (Shizuoka, Japan), were used for experiments. All mice had free access to water and standard animal food, and were exposed to a 12-h light/dark cycle (lights on between 8:00 and 20:00), a temperature of 22 ± 1°C, and a humidity-controlled environment (50 ± 5%). The mice were exposed to a low concentration of DE (DE group: n = 15) or clean air (control group: n = 15) for 8 h/day (10:00–18:00) in the same type of inhalation chambers from gestational day 2 to 17 at the Center for Environmental Health Science for the Next Generation (Research Institute for Science and Technology, Tokyo University of Science). Pregnant mice were housed in stainless steel wire mesh cages during the exposure period. Gestational day 17 is considered to be the end of the teratogenic period in mice; hence, the designated exposure period lasted from gestational day 2 to 17. After this period, dams and pups were maintained in a clean room. On postnatal day 7, the number of pups per litter was adjusted to eight by euthanasia using excess treatment of sodium pentobarbital. After weaning on postnatal day 21, male mice were maintained in groups in their home cages (four mice/cage). Body weights of dams and their pups were recorded at sampling. All samples were obtained under sodium pentobarbital (50 mg/kg) anesthesia, and all possible efforts were made to minimize the suffering of the animals.

### Locomotor activity

To investigate DE-induced locomotion changes, we used an activity monitor with an infrared ray sensor (NS-AS01; Neuroscience Inc., Tokyo, Japan) that measured spontaneous motor activity of the male offspring at 10 weeks of age by detecting the release of temperature-associated infrared rays. Each male mouse from each litter was selected to be tested for locomotion (n = 15/group). Spontaneous motor activity was counted in both the light and dark period for 1 day. Data were automatically analyzed using a computerized system (multidigital 32-port counter system; Neuroscience Inc., Tokyo, Japan).

### Social isolation-induced male territorial aggression (resident−intruder test)

The resident−intruder test was based on a previous report with slight modifications [17, 56]. Briefly, at 10 weeks of age, each male mouse from each litter was selected to be tested for aggression (n = 15/group). These mice were individually housed for 2 weeks in Plexiglas cages (30 × 20 × 12.5 cm: 7500 cm^3^) to establish a home territory and to increase the aggression of the resident experimental mice. One week prior to the encounter with the intruder mouse, the bedding in the cage was changed. Intruder male mice (similar in weight and age to the resident mice and housed in groups of four under the same conditions), which had not received any treatment, were introduced into the cage. The resident mouse was introduced into the cage first, and the intruder mouse thereafter, each at opposite corners of the box, with the wire rack for food and water removed. Territorial aggressive behavior was scored under red light for 10 min within the dark period of the light/dark cycle (between 20:00 and 1:00), including an acclimation period of 1 h before the test. Each experimental mouse was tested only once.

To minimize the possible effects of plasma corticosterone concentrations on animal behavior [57, 58] during the test period, we counterbalanced the task by controlling the order of the animals tested among the control and DE-exposed groups.

The following parameters were recorded: 1) attack latency (i.e., the time interval from the first contact to the first attack, in seconds); 2) number of attacks; 3) total time spent attacking (in seconds); and 4) number of tail rattles (a behavior typically seen prior to an attack). Videos were scored by two trained observers who remained blinded to the treatment conditions. At the end of each test, the box was cleaned with deionized water, 2% alcohol, and deionized water, and the sawdust was changed.

### Measurement of basal serum testosterone levels

Serum testosterone levels were measured according to a previously described method [22]. In brief, blood was collected between 9:00 and 10:00 on the day after the resident−intruder test. Serum samples were handled and stored at– 80°C. Detection of serum testosterone levels was performed using an enzyme-linked immunosorbent assay kit (testosterone ELISA kit, cat. no. 1880, Alpha Diagnostic Intl. Inc., San Antonio, TX, USA) according to the manufacturer’s protocol.

### Brain dissection

Brain dissection was also performed at the same time as blood collection. Brain dissection was performed according to a modified version of the method of Heffner et al. [59], and was based on the atlas described by Paxinos and Franklin [60]. The following brain regions were rapidly dissected from coronal brain sections: (1) prefrontal cortex (containing the cingulate cortex and motor cortex areas 1 and 2), (2) nucleus accumbens, (3) amygdala, (4) hypothalamus, (5) ventral tegmental area, and (6) brainstem. Levels of DA, 5-HT, and their metabolites were determined in these brain regions.

### Quantitative determination of dopamine, serotonin, and their metabolites

As described in detail previously [15, 50], levels of dopamine, serotonin, and their metabolites were quantitatively determined. Briefly, frozen brain tissues were homogenized in ice-cold 0.2 M perchloric acid (Nacalai Tesque Inc., Kyoto, Japan) containing 100 mM Na_2_EDTA (Dojindo Laboratories, Kumamoto, Japan) and 100 ng of isoprotenol (Sigma−Aldrich Co., St. Louis, MO, USA) as an internal standard. The homogenates were centrifuged at 20000 × *g* for 15 min at 0°C. Supernatants were transferred to new tubes, and the pellets were stored for a protein assay. The pH of the supernatant was adjusted to 3.5 with 1 M sodium acetate (Kanto Chemical Co., Inc., Tokyo, Japan) and stored at −80°C until required for analysis. For high-performance liquid chromatography (HPLC), 10 μL of the pH-adjusted supernatant was injected with a microsyringe (702SNR; Hamilton Co., Reno, NV, USA) into an HPLC system with electrochemical detection (HTEC-500MAB; EICOM Co., Kyoto, Japan). Each group contained samples from nine mice. The standard solution contained DA, 5-HT, and their metabolites. We analyzed the DA metabolites DOPAC and HVA, and the 5-HT metabolite 5-HIAA. Standard DA, HVA, 5-HT, and 5-HIAA were obtained from Sigma−Aldrich. Standard DOPAC was obtained from Wako Pure Chemical Industries, Ltd. (Osaka, Japan). The monoamines and their metabolites were separated by passage through a C18 reverse-phase column (Eicompak SC-5ODS; 3.0 mm × 150 mm; Eicom) maintained at 25°C, and connected to an electrochemical detector (EPC-500, Eicom). The mobile phase was 0.1 M acetic acid/citric acid buffer (pH 3.5) containing Na_2_EDTA (5 mg/L), sodium 1-octanesulfonate (190 mg/L; Nacalai Tesque), and 15% methanol (Kanto Chemical Co., Inc.). The flow rate was maintained at 0.5 mL/min for 35 min. Data were collected and analyzed using the PowerChrom 280 System (eDAQ Pty Ltd., Denistone East, Australia).

To determine protein concentrations, the pellets were dissolved in 100 mM Tris-HCl for protein determination via a highly sensitive version of the Bradford method by using a commercial reagent (ADV-01; Cytoskeleton Inc., Denver, CO, USA), and measurements were performed according to the manufacturer’s protocol. The absorbance was measured at 595 nm using a 96-well microplate reader (model 550; Bio-Rad Laboratories Inc., Hercules, CA, USA), and protein concentration was calculated from a standard curve generated with bovine gamma globulin (Pre-Diluted Protein Assay Standards: Bovine Gamma Globulin Set; Thermo Fisher Scientific Inc., Rockford, IL, USA).

### Statistical analysis

We used independent litters (1 offspring from each dam) for evaluation of behavior, neurochemical levels, and the measurement of serum testosterone levels. The independent litters comprised one pup from each dam from the control or DE-exposed groups (n = 15). Statistical analyses were performed with the independent litter as the statistical unit. These data were presented as the mean ± standard error of the mean (SEM). Student’s *t*-test was used to detect significant differences between the control and DE-exposed groups. Significance was set at P < 0.05.
